# Co-infection, reinfection and superinfection with *Anaplasma phagocytophilum* strains in a cattle herd based on *ankA* gene and multilocus sequence typing

**DOI:** 10.1186/s13071-020-04032-2

**Published:** 2020-03-30

**Authors:** Denis B. Langenwalder, Cornelia Silaghi, Marion Nieder, Martin Pfeffer, Friederike D. von Loewenich

**Affiliations:** 1 Department of Medical Microbiology and Hygiene, University of Mainz, Obere Zahlbacherstrasse 67, 55131 Mainz, Germany; 2 Institute of Infectology, Friedrich-Loeffler-Institut, Südufer 10, 17493 Greifswald-Insel Riems, Germany; 3 Institute for Animal Hygiene and Veterinary Public Health, University of Leipzig, An den Tierkliniken 1, 04103 Leipzig, Germany

**Keywords:** *Anaplasma phagocytophilum*, Variant, Cattle, *ankA*, Multilocus sequence typing (MLST), Tick-borne fever, Germany, Pathogenicity, Roe deer

## Abstract

**Background:**

*Anaplasma phagocytophilum* is a Gram-negative obligate intracellular bacterium that replicates in neutrophil granulocytes. It is transmitted by ticks of the *Ixodes ricinus* complex and causes febrile illness in humans and animals. We used multilocus sequence typing (MLST) and *ankA* gene-based typing to study the molecular epidemiology of the *A. phagocytophilum* strains circulating in a German cattle herd over one pasture season. The aim was to investigate whether co-infection with two distinct variants, reinfection with the same and/or superinfection by a different strain occurred during one pasture season. Eight genetic loci were sequenced in 47 PCR-positive samples from 15 animals.

**Results:**

Five different sequence types (ST) and four *ankA* alleles were detected in the cattle herd. Three different ST caused clinically overt tick-borne fever in primary infected animals. The concordance between ST and *ankA* allele was 100%. Therefore, the housekeeping genes used for MLST and the highly variable *ankA* gene were concatenated to increase resolution. Co-infection could be proven because samples of chronologically close collection dates were included. Co-infecting *A. phagocytophilum* strains differed by 14 to 18 single nucleotide polymorphisms (SNPs). Most superinfecting variants varied by 14 SNPs from the previous strain and appeared in median after a free interval of 31 days. Thus, it is unlikely that superinfecting strains arose by in-animal evolution. Immunity against re- or superinfection was assumed because the cattle developed clinical signs only during primary infection.

**Conclusions:**

The tick-pathogen-vertebrate host interaction is probably much more complex than previously thought taking into account the frequently occurring events of co-infection, reinfection and superinfection. This complex situation could not be easily simulated in an experimental infection and underlines the value of field studies.
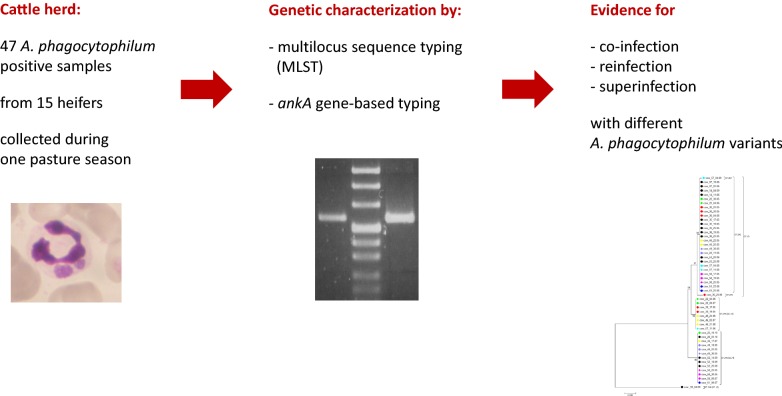

## Background

*Anaplasma phagocytophilum* is a Gram-negative obligate intracellular bacterium that replicates in neutrophil granulocytes [1]. It is transmitted by ticks of the *Ixodes ricinus* complex and causes febrile illness in humans and animals [2, 3]. The disease entity in domestic ruminants such as sheep, cattle and goats, is referred to as tick-borne fever [4]. Main clinical signs in cattle consist in fever, inappetence, cough, lower limb oedema, drop in milk yield and abortion [4, 5]. Leukopenia, thrombopenia and anemia are typical laboratory findings [6].

The incubation time to the development of clinical signs in needle-inoculated cattle varied between four to eleven days [7–9] and *A. phagocytophilum* became detectable in the blood *via* nested PCR between days 3 and 6 post-infection (pi) [6]. The maximal bacteremic phase determined by nested PCR lasted between 17–26 days pi [6]. Bovine blood obtained after day 14 pi was not infectious any more for subinoculated cattle [7]. Without treatment, clinical recovery was observed in an experimental setting in an average of eight days [6] and under field conditions within one week [10].

Outbreaks of tick-borne fever in cattle mainly happened after introduction of tick-naïve or newly bought animals to tick-infested pastures [11–14]. Further, it has been shown that the infection of cattle with *A. phagocytophilum* occurred primarily at the beginning of the pasture season [15] and especially in heifers that grazed for the first time [10]. Although cattle seem to be affected only once per lactation period, immunity appears to be short lived because attacks in the successive lactation periods might occur [7, 11]. Even if the same strains were used as a homologous challenge in an experimental setting, some of the animals were susceptible to clinically apparent tick-borne fever in the six to twelve months following the primary infection [7, 8]. However, in a recent comprehensive study of tick-borne fever in a German cattle herd over an entire pasture season of five months, five out of 15 initially infected heifers did not show clinical signs when they experienced re- or superinfection [10].

Previously, the *A. phagocytophilum* strains circulating in this herd were genetically characterized using the *16S* rRNA, *groEL*, *msp2* and *msp4* genes [10]. However, not all four loci were amplifiable in all animals due to technical problems. Therefore, each gene could only be used in terms of single locus analysis limiting the discriminatory power to distinguish distinct *A. phagocytophilum* strains. Thus, reinfection with the same strain or superinfection with a different genetic variant could not always be clearly distinguished. Further, it was unclear whether simultaneous co-infection with different genetic variants might have occurred because samples obtained at chronologically close collection dates were not considered for molecular characterization. For this reason, we chose multilocus sequence typing (MLST) [16] instead of single locus approaches and *ankA*-based typing, which has been found in the past to have high discriminatory power [17]. All positive samples still available including those from chronologically close collection dates were analyzed.

The aim was (i) to investigate whether simultaneous co-infection with different *A. phagocytophilum* strains occurs under field conditions; (ii) to examine whether reinfection with the same strain takes place during one pasture season; (iii) to study whether superinfection with a second variant happens during the same pasture period; and (iv) to observe whether reinfection or superinfection lead to clinical signs.

Cattle on pasture are continuously exposed to ticks. Therefore, it is impossible in field studies to clearly distinguish between reinfection with the same strain or reactivation of latent infection. Similarly, it is impractical to definitely separate in-animal evolution of a variant from superinfection. Nevertheless, the results of the genetic characterization presented here and the findings from previous experimental infections helped to define criteria that are discriminatory at least to a certain extent. Here, we report the circulation of six *A. phagocytophilum* variants in varying combinations in a cattle herd over one pasture season. This complex situation could not be easily simulated in an experimental infection and underlines the value of field studies.

## Methods

### Samples

The blood samples were collected from a dairy cattle herd in North-Rhine-Westphalia, Germany as part of a study reported previously [10]. The stock consisted of 39 cows and 19 heifers (*Bos taurus*). The term ‘heifer’ is used synonymously for ‘first calf heifer’ throughout the article. The animals were cross-breeds of Red and Black Holstein Friesian and German Simmental in a closed breeding system. This implies that none of the animals was bought in addition from other farmers. The heifers went to the pasture for the first time, whereas the cows had grazed during the previous seasons. The samples investigated here were from the 15 heifers that tested positive for *A. phagocytophilum* DNA between May and October 2011 [10]. In total, 47 samples from different collections dates were included. Generally, blood samples of each animal were taken prior to pasturing in May 2011 and then every other month until January 2012. After an animal was PCR-positive, blood samples were taken weekly for the following 6–8 weeks and thereafter every other week for further 6–8 weeks. The rectal temperature was measured daily and a value of ≥ 39.5 °C was considered as fever. Blood samples were drawn weekly in febrile animals. Further, the heifers were observed by the farmer for reduced milk yield, discharge from eyes and nose, lower limb oedema and stiff walking.

### Sequencing

DNA from EDTA-anticoagulated blood samples was extracted using the High Pure PCR Template Preparation Kit (Roche, Mannheim, Germany). Seven housekeeping genes (*pheS*, *glyA*, *fumC*, *mdh*, *sucA*, *dnaN* and *atpA*) were used for MLST as reported previously [16]. Clonal complexes (CC) were defined by sharing identical alleles at five of the seven loci with at least one other member of the group. The *ankA* gene was partially amplified and bidirectionally sequenced as described [16]. GenBank accession numbers are shown in Additional file 1: Table S1. The MLST profiles for samples without ambiguous nucleotides were submitted to the *A. phagocytophilum* isolates database hosted on PubMLST (https://pubmlst.org/aphagocytophilum/).

### Definition of persistence, co-infection, reinfection and superinfection

The maximal bacteremic phase in experimentally infected cattle lasted between 17–26 days pi as determined by nested PCR [6]. Therefore, the continuous presence of the same ST in consecutive samples within 30 days after the first positive PCR was interpreted as persistence of the initially infecting *A. phagocytophilum* strain. The co-infection with different *A. phagocytophilum ankA* variants has been described in a bovine congenital infection [18]. Therefore, the presence of different ST in consecutive samples after the first positive PCR was defined as co-infection with distinct *A. phagocytophilum* strains. Reinfection with the same ST was considered when the same ST reoccurred later than 30 days after the primary infection and at least two negative PCR results were obtained in between. Superinfection with a new ST was supposed when it was detected after at least one negative PCR result.

### Phylogenetic analysis

Sequences were codon-aligned by ClustalW applying the PAM (Dayhoff) matrix. Trees were constructed using the neighbor-joining (NJ) method with the Jukes-Cantor model and the complete deletion option in the program MEGA X version 10.0.5 [19]. Bootstrap analysis was conducted with 1000 replicates. Pairwise distances between the sequences were computed using the Jukes-Cantor matrix and applying the complete deletion option.

### Comparison of typing methods

Adjusted Wallace coefficients [20] and Simpson’s index of diversity [21] were calculated using the online tool available at: http://www.comparingpartitions.info/index.php?link=Tool.

## Results

### Cow samples

In total, 47 samples of different collection dates from 15 heifers were included. Animals were positive for *A. phagocytophilum* DNA between one to six times (Table 1). One heifer (cow 7) was not examined for clinical signs. All others, except one (cow 59), suffered at the time of the first positive PCR result from clinically apparent tick-borne fever. The animals were not specifically treated against the infection and recovered in average within one week [10]. Heifers remained asymptomatic after the first detection of *A. phagocytophilum* by PCR even if they experienced second infection.

**Table 1:** Results of the genetic characterization of the *A. phagocytophilum* strains infecting the 15 heifers

Cow no.	Date of positive PCR	ST	*ankA* allele	Days between positive PCR results^a^	Days between positive PCR results^b^	Interpretation
7	19.06.2011	243	a	na	na	na
	25.06.2011	243	a	6	6	Persistence
14	04.06.2011	243	a	na	na	na
	11.06.2011	243	a	7	7	Persistence
22	30.05.2011	243	a	na	na	na
	04.06.2011	243, 244	a, b	5	5	Persistence, co-infection
	09.07.2011	244	b	40	35	Reinfection
	16.10.2011	245	c	139	99	Superinfection
28	03.10.2011	245	c	na	na	na
30	17.05.2011	244	b	na	na	na
	19.05.2011	244	b	2	2	Persistence
	25.05.2011	243	a	8	6	Co-infection
	30.05.2011	243	a	13	5	Persistence
	04.06.2011	243	a	18	13	Persistence
	25.06.2011	275	a	39	21	Superinfection
35	17.05.2011	243	a	na	na	na
	19.05.2011	243	a	2	2	Persistence
	25.05.2011	243	a	8	6	Persistence
36	19.05.2011	243	a	na	na	na
	25.05.2011	243	a	6	6	Persistence
46	22.05.2011	243	a	na	na	na
	25.05.2011	243	a	3	3	Persistence
	25.06.2011	244	b	34	31	Superinfection
	03.07.2011	244	b	42	8	Persistence
	17.07.2011	245	c	56	14	Superinfection
	21.08.2011	244	b	91	35	Reinfection
49	19.05.2011	245	c	na	na	na
	25.05.2011	245	c	6	6	Persistence
	30.05.2011	243, 245	a, c	11	5	Persistence, co-infection
	11.06.2011	243	a	23	12	Persistence
52	14.09.2011	245	c	na	na	na
	18.09.2011	245	c	4	4	Persistence
	25.09.2011	245	c	11	7	Persistence
53	22.06.2011	243	a	na	na	na
	25.06.2011	243	a	3	3	Persistence
57	04.06.2011	243	a	na	na	na
	11.06.2011	243, 244	a, b	7	7	Persistence, co-infection
	04.09.2011	297	a	92	85	Superinfection
58	17.05.2011	243	a	na	na	na
	19.05.2011	243	a	2	2	Persistence
	25.05.2011	243, 245	a, c	8	6	Persistence, co-infection
	30.05.2011	245	c	13	5	Persistence
	09.07.2011	245	c	53	40	Reinfection
59	04.09.2011	246	d	na	na	na
61	22.06.2011	243	a	na	na	na
	25.06.2011	243	a	3	3	Persistence
	09.07.2011	245	c	17	14	Co-infection

^a^Referring to the first positive PCR result

^b^Referring to the previous positive PCR result

*Abbreviations*: na, not applicable

### MLST of the *A. phagocytophilum* strains infecting the cows

In general, different sequences of a given locus (*pheS*, *glyA*, *fumC*, *mdh*, *sucA*, *dnaN*, *atpA*) were ascribed a unique, but arbitrary allele number and each unique combination of alleles was assigned a sequence type (ST). Six different ST were found to infect the cattle herd (Table 1). In 9% (4/47) of the samples, ambiguous nucleotides were found in three to four loci. The ambiguous nucleotides could be resolved, because three of the animals (cow 22, cow 49 and cow 58) harbored the respective other ST before and after the time of the double infection, although formally the PCR products were sequenced directly without prior cloning. The nucleotide exchanges in the animals simultaneously harboring different ST are shown in Additional file 2: Table S2a and the ascribed allele numbers in Additional file 2: Table S2b.

**Fig. 1:**
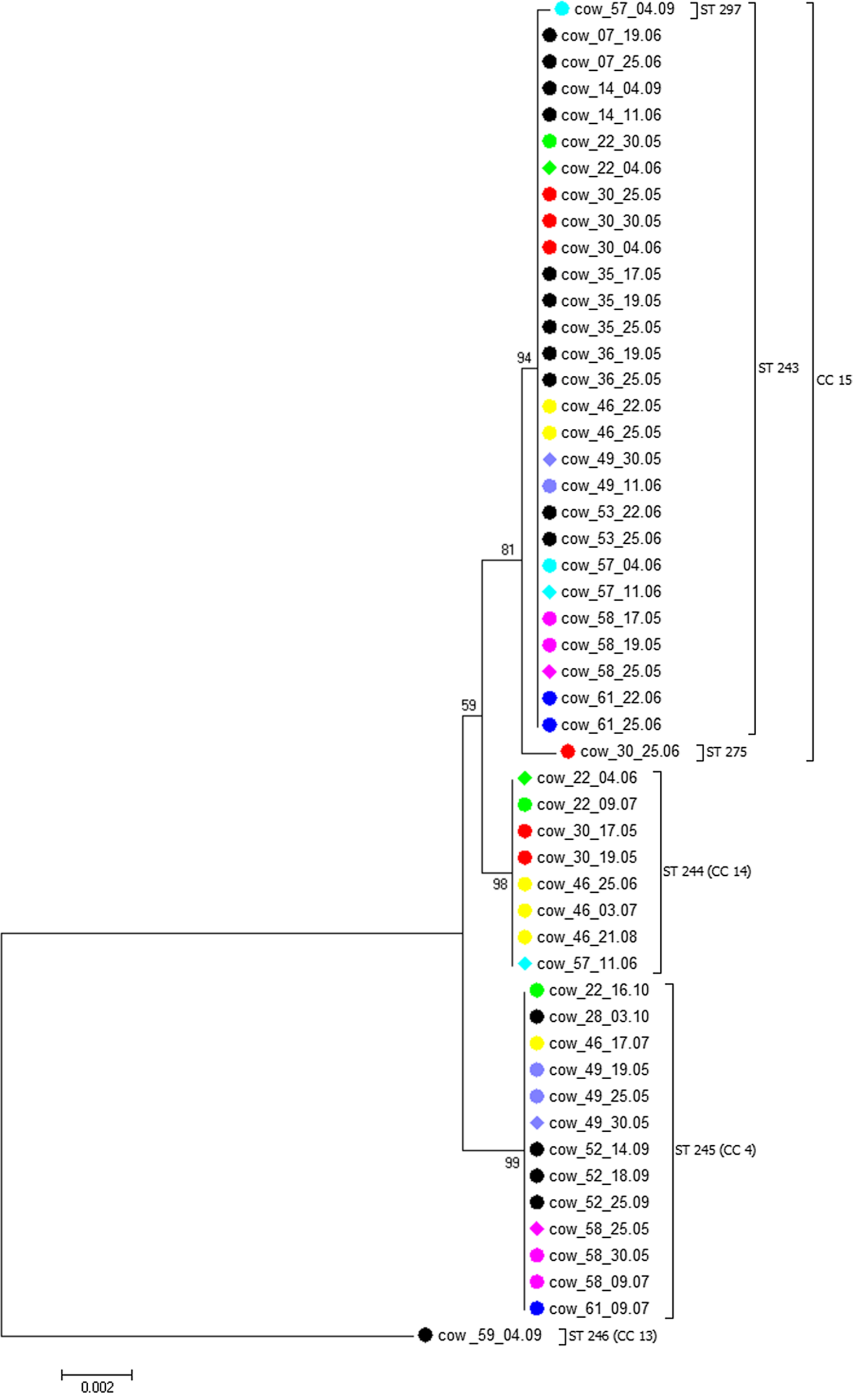
Neighbour-joining (NJ) phylogenetic tree calculated from the concatenated housekeeping gene sequences. Tree construction was achieved by the NJ method using the Jukes-Cantor matrix with the complete deletion option. Bootstrap values are shown next to the branches. The scale-bar indicates the number of nucleotide substitutions per site. The final data set contained 2877 positions. Animals infected with only one ST are symbolized by black circles. Those harboring more than one ST over time are represented by green, red, yellow, purple, light blue, pink and dark blue circles. Ambiguous nucleotides in heifers with different ST at the same collection date were resolved before tree construction. Animals infected simultaneously with two different ST are represented by diamonds at the respective time point

ST 297 was a single locus variant (SLV) and ST 275 a double locus variant (DLV) of ST 243. Together, this three ST formed the new clonal complex (CC) 15 (Fig. 1). The novel CC 14 was constituted by ST 244 and by ST 21 which was formerly found in a European bison (*Bison bonasus*) [16]. ST 245 was part of CC 4 that has been described to contain samples from European bison and goat (*Capra aegagrus hircus*) [16]. Cow 59, which never developed clinical tick-borne fever, harbored ST 246 that belonged to the recently defined CC 13. CC 13 was found to include samples from roe deer (*Capreolus capreolus*) and water buffalo (*Bubalus bubalis*) [22].

The concatenated nucleotide sequences (2877 bp) of ST 243, ST 244 and ST 245 differed by 4–13 single nucleotide polymorphisms (SNPs) and were highly identical to each other (Table 2). ST 297 infecting cow 57 on the 4th of September varied only by one SNP in the *sucA* locus from ST 243. However, the roe deer-associated ST 246 found in the asymptomatic cow 59 was more distantly related. It showed 76 to 78 SNPs when compared to the other five cattle-associated ST.

**Table 2:** SNPs (below the diagonal) and percent identity (above the diagonal) between the concatenated housekeeping gene sequences of the six ST infecting the cattle herd

	ST 243	ST 244	ST 245	ST 246	ST 275	ST 297
ST 243		99.76	99.62	97.31	99.86	99.97
ST 244	7		99.72	97.35	99.69	99.72
ST 245	11	8		97.35	99.55	99.58
ST 246	77	76	76		97.31	97.27
ST 275	4	9	13	77		99.83
ST 297	1	8	12	78	5	

### *ankA* gene-based typing of the *A. phagocytophilum* strains infecting the cows

**Fig. 2:**
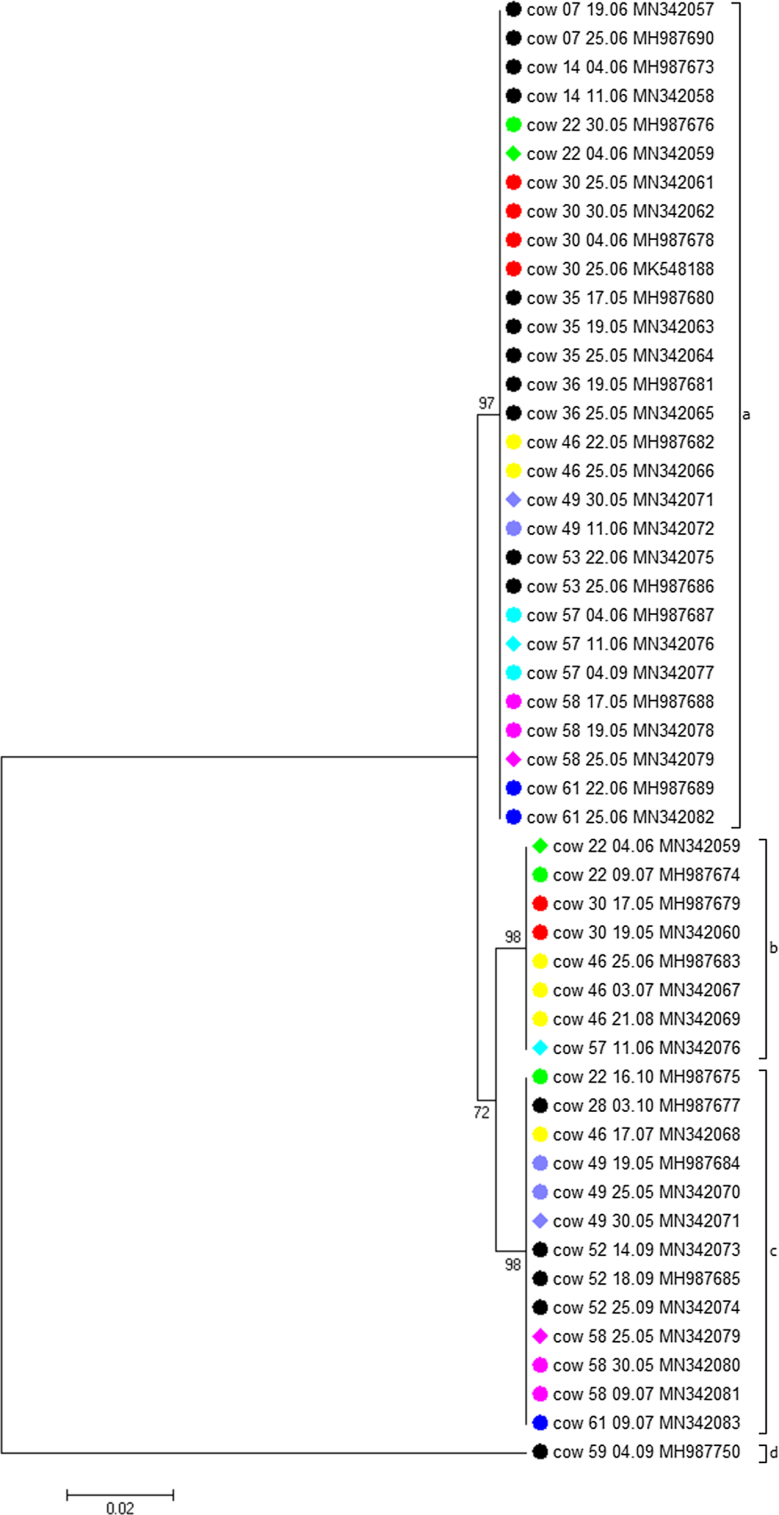
Neighbour-joining (NJ) phylogenetic tree calculated from the *ankA* gene sequences. Tree construction was achieved by the NJ method using the Jukes-Cantor matrix with the complete deletion option. Bootstrap values are shown next to the branches. The scale-bar indicates the number of nucleotide substitutions per site. The final data set contained 520 positions. GenBank accession numbers are given after the sample designation. Animals infected with only one *ankA* allele are symbolized by black circles. Those harboring more than one *ankA* allele over time are represented by green, red, yellow, purple, light blue, pink and dark blue circles. Ambiguous nucleotides in heifers with different *ankA* allele at the same collection date were resolved before tree construction. Animals infected simultaneously with two different *ankA* alleles are represented by diamonds at the respective time point

Four different *ankA* alleles were found to infect the cattle herd (Table 1). They were designated with letters (a, b, c, d) to prevent confusion with the *ankA* gene clusters described earlier [16]. *ankA* allele a had the highest prevalence with 53% (25/47) (Fig. 2). In 9% (4/47) ambiguous nucleotides were present in the *ankA* chromatograms. These were the same heifers that were simultaneously infected with two *A. phagocytophilum* ST at the respective time point (Additional file 2: Table S2). The ambiguous nucleotides could be resolved because three animals (cow 22, cow 49 and cow 58) harbored the respective other *ankA* allele before and after the time of the double infection, although formally the PCR products were sequenced directly without prior cloning.

The *ankA* sequences (520 bp) of alleles a, b and c differed by 6–7 SNPs and were 99% identical to each other (Table 3). However, *ankA* allele d found in the asymptomatic cow 59 was more distantly related. It showed 100 to 102 SNPs (without gaps) and had an identity of 80–81% when compared to the other three cattle-associated *ankA* alleles.

**Table 3:** SNPs (below the diagonal) and percent identity (above the diagonal) between *ankA* allele sequences of the four *ankA* alleles found in the cattle herd

	a	b	c	d
a		98.64	98.64	80.82
b	7		98.84	80.32
c	7	6		80.32
d	100	102	102	

### Concordance between typing methods

Adjusted Wallace coefficients [20] were calculated to test for concordance between the different typing methods. The concordance between ST, CC and *ankA* allele was 100% when the 43 samples without ambiguous nucleotides were considered. The concordance between *ankA* and ST was still high (> 75%), but limited to 80% because ST 243, ST 275 and ST 297 shared *ankA* allele a (Table 4).

**Table 4:** Adjusted Wallace coefficients for ST, CC, *ankA* allele and *msp4* variant

	ST	CC	*ankA*	*msp4*
ST		1.000 (1.000–1.000)	1.000 (1.000–1.000)	1.000 (1.000–1.000)
CC	0.802 (0.561–1.000)		1.000 (1.000–1.000)	1.000 (1.000–1.000)
*ankA*	0.802 (0.561–1.000)	1.000 (1.000–1.000)		1.000 (1.000–1.000)
*msp4*	0.772 (0.411–1.000)	1.000 (1.000–1.000)	1.000 (1.000–1.000)	

*Notes*: Only samples without ambiguous nucleotides were considered. The 95% confidence intervals are given in parentheses

Earlier, the *A. phagocytophilum* strains circulating in the cattle herd were genetically characterized using the *16S* rRNA, *groEL*, *msp2* and *msp4* genes [10]. However, not all loci were amplifiable in all samples and due to technical problems, some of the sequence reads were not of full length. Only two *16S* rRNA and two *groEL* gene variants were reported [10]. Therefore, the concordance between *16S* rRNA (< 10%) and *groEL* (55.5%) gene-based typing and ST, CC and *ankA* allele was low (data not shown). Two *msp2* variants were found previously in the cattle herd [10]. However, a full data set was only comparable in 11 samples yielding a concordance between *msp2*-based typing *versus* ST, CC and *ankA* allele of 100% (data not shown). Four *msp4* types [10] from 19 samples could be used for comparison. The concordance between *msp4 versus* CC and *ankA* allele was 100% (Table 4). The concordance between *msp4* and ST was still high (> 75%), but limited to 77% because *msp4* variant M4-49 [10] was shared by ST 243 and ST 297 (differing by one SNP in the *sucA* locus).

Simpson’s index of diversity [21] was calculated as indication of the power of a typing method to distinguish between unrelated strains. It was determined in the 43 samples without ambiguous nucleotides. The Simpson’s index of diversity was highest for ST, but this was not statistically significant because of overlapping 95% confidence intervals (Table 5).

**Table 5:** Simpson’s index of diversity calculated using the 43 samples from the cattle herd without ambiguous nucleotides

	Partitions	Simpson’s index of diversity
ST	6	0.642 (0.530–0.754)
CC	4	0.590 (0.473–0.707)
*ankA*	4	0.590 (0.473–0.707)

*Notes*: The 95% confidence intervals are given in parentheses

### Persistence

Our definition of persistence, co-infection, reinfection and superinfection is explained in the methods section. Six animals (cow 7, cow 14, cow 35, cow 36, cow 52 and cow 53) were infected with the same ST over time (Additional file 3: Table S3). The median presence of an identical ST in the blood was 7 days and the maximum presence 12 days.

### Co-infection

The *ankA* gene probably undergoes recombination [23]. Therefore, it could bias the phylogenetic analysis and was not concatenated with the housekeeping genes used for MLST [16]. Here, the concordance between ST and *ankA* allele was 100% (Table 4). Therefore, the concatenation of the housekeeping genes with *ankA* did not disturb the interpretation and allowed the comparison of a longer sequence fragment.

Simultaneous co-infection with two ST occurred in six animals (cow 22, cow 30, cow 49, cow 57, cow 58 and cow 61). The co-infecting *A. phagocytophilum* strains differed by 14–18 SNPs when the 3397 bp (without gaps) of the concatenated housekeeping and *ankA* gene sequences were considered. The co-infecting strains were detected within a median interval of 6 days (Additional file 3: Table S3).

### Reinfection

Three heifers (cow 22, cow 46 and cow 58) were reinfected with the same ST 35–40 days after the last positive PCR result (Additional file 3: Table S3). Heifers were positive by PCR only once when they acquired reinfection.

### Superinfection

Superinfection with a new ST was observed in four heifers (cow 22, cow 30, cow 46 and cow 57) after a median free interval of 31 days after the last positive PCR result (Additional file 3: Table S3). Three negative PCR results were obtained in median between the detection of the previous *A. phagocytophilum* strain and the superinfecting variant. Cow 46 was superinfected twice with different ST (Additional file 3: Table S3). The 3397 bp (without gaps) of the concatenated housekeeping and *ankA* gene sequences of the superinfecting strains differed by one (ST 297), four (ST 275) or 14 (ST 244, ST 245) SNPs from the variant detected before. Except for cow 46, which experienced superinfection with ST 244 lasting 8 days (Additional file 3: Table S3), heifers were positive by PCR only once when they acquired superinfection.

### Comparison to previously published housekeeping gene sequences

The concatenated housekeeping gene sequences from our study were compared to 347 sequences without ambiguous nucleotides described previously [16, 22, 24–26]. The sequences were found in six clusters (Additional file 4: Fig. S1). Clusters 1, 2 and 3 were described earlier [16]. Cluster 1 contained sequences from humans, domestic animals (dogs, horses and cats), farm animals (cattle, sheep and goats), large wild animals (wild boars, European bison, red deer, chamois and red foxes), small mammals (hedgehogs) and *I. ricinus* ticks. Cluster 2 harbored samples from roe deer and *I. ricinus* ticks, but also sporadically sequences from domestic ruminants (three goats, one water buffalo and the asymptomatically infected cow 59). Cluster 3 was restricted to strains from rodents and shrews. The newly described Cluster 4 contained only three sequences (two roe deer and one red deer). Cluster 5 and Cluster 6 were originally described to be separate clades of Cluster 1 and 3, respectively [25] and harbored exclusively *I. persulcatus* and *I. pavlovskyi* ticks from the Asian part of Russia.

### Comparison to previously published *ankA* gene sequences

The *ankA* sequences from our study were compared to 389 sequences without ambiguous nucleotides described previously [16, 22, 26]. The sequences were found in five clusters (Additional file 5: Fig. S2). described earlier [16]. Cluster 1 contained sequences from humans, domestic animals (dogs, horses and cats), farm animals (cattle, sheep and goats), large wild animals (wild boars, European bison, red deer, chamois and red foxes), small mammals (hedgehogs) and *I. ricinus* ticks. Cluster 2 harbored samples from roe deer and *I. ricinus* ticks, but also sporadically sequences from domestic ruminants (one goat, one water buffalo and the asymptomatically infected cow 59). Cluster 3 was, with the exception of two red deer strains, restricted to samples from roe deer. Cluster 4 contained strains from domestic ruminants (cattle, sheep and goats), wild ruminants (red deer, European bison, roe deer and chamois) and *I. ricinus* ticks. Cluster 5 was restricted to samples from rodents and shrews.

## Discussion

MLST and *ankA*-based typing were proven to be suitable to distinguish the *A. phagocytophilum* strains circulating in a German cattle herd over one pasture season because six ST and four *ankA* alleles were detected. Both methods had high discriminatory power (Table 5). In contrast to the former attempt [10], all eight loci were amplifiable and of full length. Previously, two *16S* rRNA, two *groEL*, two *msp2* and four *msp4* variants were found in the herd [10]. Thus, only *msp4*-based typing was of comparable usefulness. However, single locus typing has an inferior resolution than multilocus typing [27]. The advantage of our MLST scheme is that it combines a standardized approach, a universe nomenclature and a free data exchange *via* the internet (https://pubmlst.org/aphagocytophilum/).

The maximal bacteremic phase in experimentally infected cattle determined by nested PCR lasted between 17–26 days pi [6]. Considering an incubation time of 4–11 days [7–9], this is in line with our finding that the median presence of an identical *A. phagocytophilum* strain in the blood was seven days and the maximum presence 12 days.

Co-infection with two strains occurred in six animals. Their concatenated housekeeping and *ankA* gene sequences showed 14–18 SNPs. It seems unlikely that such an amount of SNPs arose *via* in-animal evolution because the new variant was detected within a median interval of six days after the first one. In sheep experimentally infected simultaneously with two different *16S* rRNA gene variants, both of them were detectable in their blood at day 0 [28]. Thereafter, depending on the proportion of both variants in the inoculum, only one of them was seen in peripheral blood. The second variant reappeared between four to 31 days pi. Thus, the initial detection of only one of both strains at the time of clinical presentation does not exclude co-infection. Formally, superinfection with a second variant within a medial interval of six days cannot be ruled out, although this seems less likely. The concept of co-infection with different *A. phagocytophilum* variants is supported by the fact that 41% (14/34) of *I. ricinus* ticks were infected with more than one ST [16]. Further, naturally infected cattle simultaneously harbored more than one *A. phagocytophilum* variant because double peaks were observed in the chromatograms of the genetic loci investigated [26, 29]. However, most animals were only sampled once, or the full profile of all loci was not available in these studies.

Reinfection with the same *A. phagocytophilum* strain occurred 35–40 days after the last positive PCR result. Cattle experimentally infected with *A. phagocytophilum* achieved pathogen clearance within 14 days as bovine blood obtained thereafter was not infectious any more for sub-inoculated cattle [7]. Thus, in our setting, reinfection seems to be more probable than reactivation of latent infection, although it is impossible in a field study to clearly distinguish between both of them. In contrast to cattle, needle-inoculated sheep housed under tick-free conditions experienced recurrent asymptomatic cycles of bacteremia after recovering from clinically overt tick-borne fever [30]. The average negative period between two of these consecutive cycles was five days.

The concatenated housekeeping and *ankA* gene sequences of the superinfecting strains differed in most instances by 14 SNPs from the variant detected before. It is unlikely that this amount of SNPs arose by in-animal evolution. However, the single SNP that distinguishes ST 243 and the superinfecting ST 297 might have arisen by recent microevolution. The superinfection with a new *A. phagocytophilum* variant has been observed before in four cattle that became PCR-positive again after an interval of 7–17 months [29]. However, it was not reported whether they experienced a second attack of clinically apparent tick-borne fever. Further, in contrast to our study, the animals were treated against tick-borne fever with oxytetracycline [29] probably biasing the results.

After the first attack, the heifers studied here were immune against clinically overt tick-borne fever for the entire pasture season of five months [10]. Accordingly, cattle were affected under field conditions only once per lactation period [7]. This is in contrast to the experimental setting where 11/14 [7] or 2/5 [8] animals were susceptible to clinically apparent tick-borne fever in the six to twelve months following the primary infection. The microscopic re-occurrence of *A. phagocytophilum* was reported in one of these two studies that both were done before PCR became available [8]. However, it is so far unknown whether the clinically immune animals experienced asymptomatic reinfection because only symptomatic cattle were investigated by blood smear. On the other hand, the microscopic detection of *A. phagocytophilum* is mostly associated with clinically apparent infection [10]. One explanation for the discrepancy between field and experimental conditions might be the fact that cattle on pasture are continuously re-exposed to the pathogen by what immunity is probably boosted.

Immunity was not sterile in the cattle herd studied here because animals were reinfected with the same or superinfected by a different strain. With one exception, the PCR was positive for only one further time in the seven other events of re- or superinfection. This could reflect semi-immunity that shortens the bacteremic phase and protects against clinically apparent tick-borne fever.

Experimentally infected cattle were clinically immune against a homologous bovine *A. phagocytophilum* strain when challenged within three months after the primary infection [31]. This was not the case when a heterologous cattle strain was used. However, we observed under field conditions protection against clinically overt tick-borne fever when superinfection with a heterologous variant occurred. It is unclear so far which degree of genetic relatedness would be sufficient to confer cross-protection because the strains used previously were not characterized genetically [31].

One of the heifers did not develop apparent tick-borne fever at all. Its concatenated housekeeping and *ankA* sequences differed by 177–178 SNPs from all other strains. The respective variant was found together with roe deer samples in the phylogenetic analysis based on MLST (Cluster 2) and *ankA* (Cluster 2) (Figs. 3, 4). It could be argued that roe-deer associated strains are less pathogenic for domestic ruminants because a similar observation was made previously in an asymptomatically infected goat [22]. However, an *A. phagocytophilum* strain from a water buffalo with typical signs of tick-borne fever was also found in the roe-deer-associated MLST and *ankA* gene Cluster 2 [22]. On the other hand, 98% (62/63) available bovine housekeeping gene sequences from this and previous studies [16, 26] were part of MLST Cluster 1. Further, 82% (58/71) of the *ankA* sequences from cattle were found in *ankA* Cluster 1 and 17% (12/71) in Cluster 4. Using a multilocus sequence approach, a similar observation was made [32]. Samples from cattle were found in three clusters. Two of them contained the majority of bovine sequences, whereas the third harbored all roe deer samples and only three genotypes from cattle. Thus, it was claimed that roe deer are probably not the reservoir host for bovine tick-borne fever [16, 32].

We describe here two new MLST clusters (5 and 6) that were originally published to be separate clades of Cluster 1 and Cluster 3, respectively [25]. They harbored exclusively *I. persulcatus* and *I. pavlovskyi* ticks from the Asian part of Russia. A similar observation was made recently as *groEL* haplotypes from *I. persulcatus* from Russia were found in the segregated *groEL* Cluster 4 [33].

## Conclusions

To the best of our knowledge, we report for the first time a comprehensive molecular characterization of the *A. phagocytophilum* strains circulating in a cattle herd over one pasture season and provide evidence for the occurrence of co-infection, reinfection and superinfection. This was only possible, because, in contrast to previous studies, samples of close collection dates were included. Further, our analysis highlights that the tick-pathogen-vertebrate host interaction is much more complex than previously thought. Whole blood from naturally infected animals was often used as inoculum in previous experimental infections. The respective *A. phagocytophilum* strains were not characterized at all [31] or only be means of *16S* rRNA gene-based typing [28]. The low discriminatory power of the *16S* rRNA gene and the fact that field samples may contain not only a single *A. phagocytophilum* variant, limits the conclusions drawn from such studies. Therefore, a reliable and easily comparable typing method such as MLST should be preferably used for genetic characterization.

## Supplementary information

**Additional file 1: Table S1.** GenBank accession numbers of the housekeeping and *ankA* gene sequences from the 47 samples from the 15 heifers.

**Additional file 2: Table S2. a** Nucleotide exchanges in five of the seven housekeeping genes (*pheS, glyA, fumC, sucA, dnaN*) and the *ankA* gene found in the four animals with simultaneous double infection with two different *A. phagocytophilum* strains. **b** Allel designation and ST found in the four animals with simultaneous double infection with two different *A. phagocytophilum* strains.

**Additional file 3: Table S3.** Detection of *A. phagocytophilum* by PCR for all collection dates in the 15 heifers and results of the genetic characterization.

**Additional file 4: Figure S1.** Neighbour-joining (NJ) phylogenetic tree calculated from the concatenated housekeeping gene sequences of the 43 cattle samples without ambiguous nucleotides and 347 samples without ambiguous nucleotides described previously. Tree construction was achieved by the NJ method using the Jukes-Cantor matrix with the complete deletion option. Bootstrap values ≥ 86% are shown. The scale-bar indicates the number of nucleotide substitutions per site. The final data set contained 2877 positions. Identical ST are displayed only once per species. The number in parenthesis indicates the frequency with which the respective ST was found. *Key*: red circles, sequences from humans, dogs, horses and cats; dark blue diamonds, sequences from domestic ruminants; light blue diamonds, sequences from wild ruminants; green triangles, sequences from small mammals; yellow squares, sequences from wild boars; purple triangles, sequences from red foxes; white triangles, sequences from ticks.

**Additional file 5: Figure S2.** Neighbour-joining (NJ) phylogenetic tree calculated from the *ankA* gene sequences of the 43 cattle samples without ambiguous nucleotides and 389 samples without ambiguous nucleotides described previously. Tree construction was achieved by the NJ method using the Jukes-Cantor matrix with the complete deletion option. Bootstrap values ≥ 81% are shown. The scale-bar indicates the number of nucleotide substitutions per site. The final data set contained 516 positions. Identical *ankA* sequences are displayed only once per species. GenBank accession numbers are given after the species designation. The number in parenthesis indicates the frequency with which the respective sequences was found. *Key*: red circles, sequences from humans, dogs, horses and cats; dark blue diamonds, sequences from domestic ruminants; light blue diamonds, sequences from wild ruminants; green triangles, sequences from small mammals; yellow squares, sequences from wild boars; purple triangles, sequences from red foxes; white triangles, sequences from ticks.

## Data Availability

All data generated or analysed during this study are included in this published article and its additional files. All newly generated nucleotide sequences were submitted to the GenBank database under the accession numbers shown in Additional file 1: Table S1. The MLST profiles for samples without ambiguous nucleotides were submitted to the *A. phagocytophilum* isolates data base hosted on PubMLST (https://pubmlst.org/aphagocytophilum/).

## Abbreviations

CC: clonal complex
MLST: multilocus sequence typing
J: neighbor-joining
SNP: single nucleotide polymorphism
ST: sequence type
