# Supplementary figures and images for: Co-infection, reinfection and superinfection with Anaplasma phagocytophilum strains in a cattle herd based on ankA gene and multilocus sequence typing
Source: Parasit Vectors. 2020 Mar 30;13:157. doi: 10.1186/s13071-020-04032-2 (PMC7106686; doi:10.1186/s13071-020-04032-2)

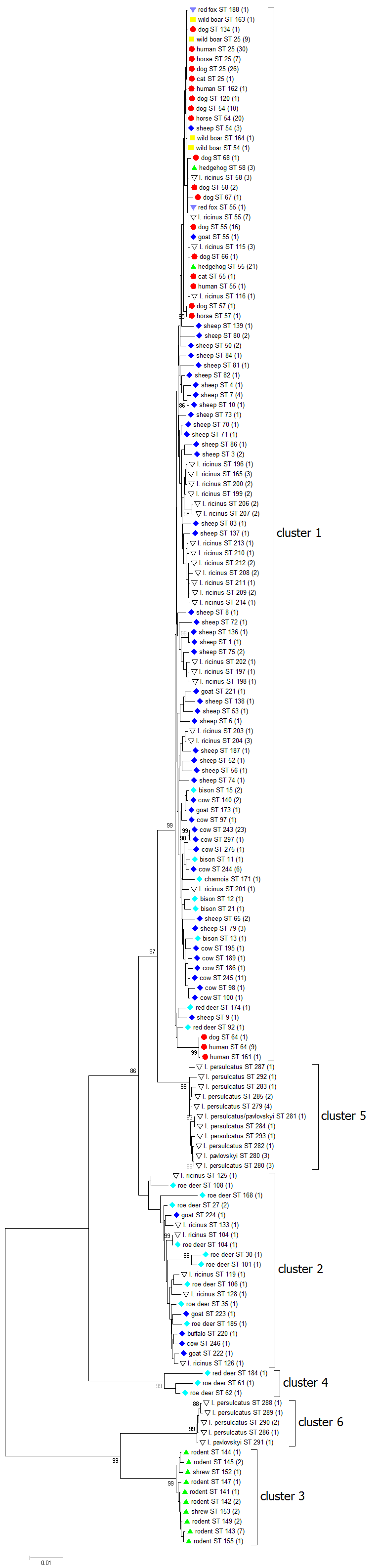

Supplement: Supplementary file 4 — Additional file 4: Figure S1. Neighbour-joining (NJ) phylogenetic tree calculated from the concatenated housekeeping gene sequences of the 43 cattle samples without ambiguous nucleotides and 347 samples without ambiguous nucleotides described previously. Tree construction was achieved by the NJ method using the Jukes-Cantor matrix with the complete deletion option. Bootstrap values ≥ 86% are shown. The scale-bar indicates the number of nucleotide substitutions per site. The final data set contained 2877 positions. Identical ST are displayed only once per species. The number in parenthesis indicates the frequency with which the respective ST was found. Key: red circles, sequences from humans, dogs, horses and cats; dark blue diamonds, sequences from domestic ruminants; light blue diamonds, sequences from wild ruminants; green triangles, sequences from small mammals; yellow squares, sequences from wild boars; purple triangles, sequences from red foxes; white triangles, sequences from ticks. [file 13071_2020_4032_MOESM4_ESM.tif]

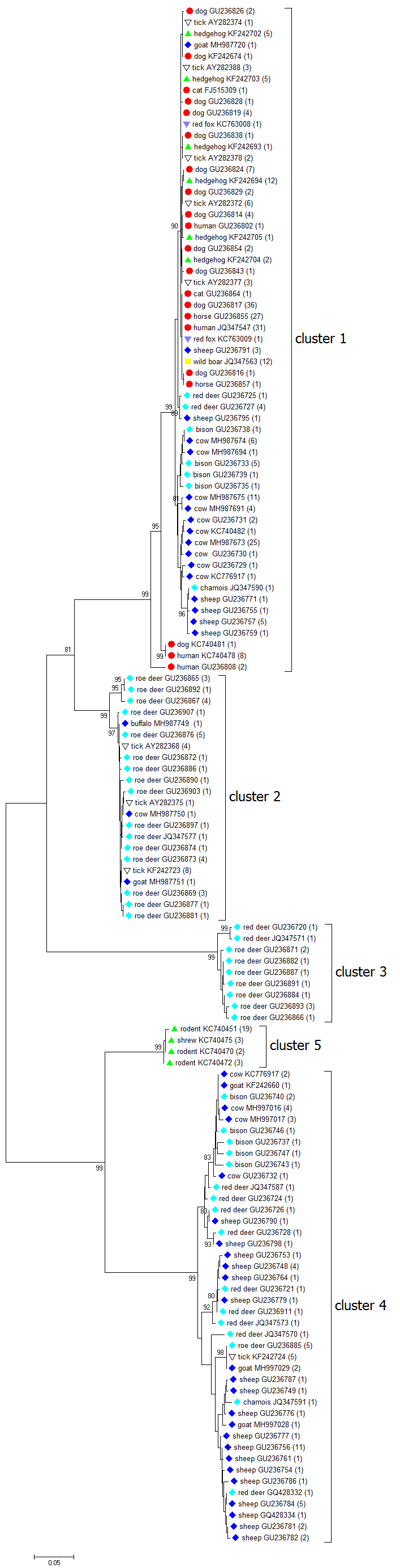

Supplement: Supplementary file 5 — Additional file 5: Figure S2. Neighbour-joining (NJ) phylogenetic tree calculated from the ankA gene sequences of the 43 cattle samples without ambiguous nucleotides and 389 samples without ambiguous nucleotides described previously. Tree construction was achieved by the NJ method using the Jukes-Cantor matrix with the complete deletion option. Bootstrap values ≥ 81% are shown. The scale-bar indicates the number of nucleotide substitutions per site. The final data set contained 516 positions. Identical ankA sequences are displayed only once per species. GenBank accession numbers are given after the species designation. The number in parenthesis indicates the frequency with which the respective sequences was found. Key: red circles, sequences from humans, dogs, horses and cats; dark blue diamonds, sequences from domestic ruminants; light blue diamonds, sequences from wild ruminants; green triangles, sequences from small mammals; yellow squares, sequences from wild boars; purple triangles, sequences from red foxes; white triangles, sequences from ticks. [file 13071_2020_4032_MOESM5_ESM.tif]
